# SFX analysis of non-biological polycrystalline samples

**DOI:** 10.1107/S2052252515002146

**Published:** 2015-03-20

**Authors:** Tao Zhang, Shifeng Jin, Yuanxin Gu, Yao He, Ming Li, Yang Li, Haifu Fan

**Affiliations:** a Institute of Physics, Chinese Academy of Sciences, No. 8, South 3rd Street, Zhong Guan Cun, Beijing, 100190, People’s Republic of China; b Program in Cellular and Molecular Medicine, Boston Children’s Hospital, Harvard Medical School, 3 Blackfan Circle, Boston, MA 02115, USA

**Keywords:** serial crystallography, X-ray powder diffraction, phase identification, structure solution

## Abstract

Serial femtosecond crystallography (SFX) is capable of collecting three-dimensional single-crystal diffraction data using polycrystalline samples. This may dramatically enhance the power of X-ray powder diffraction. In this paper a test has been performed using simulated diffraction patterns. The test sample is a mixture of two zeolites with crystal grain sizes from 100 to 300 nm. X-ray diffraction snapshots by SFX were simulated and processed using the program suite *CrystFEL*. Identification according to the primitive unit-cell volume determined from individual snapshots was able to separate the whole set of snapshots into two subsets, which correspond to the two zeolites in the sample. Monte Carlo integration in *CrystFEL* was then applied to them separately. This led to two sets of three-dimensional single-crystal diffraction intensities, based on which crystal structures of the two zeolites were solved easily by direct methods implemented in the program *SHELXD*.

## Abstract 2

A serial femtosecond crystallography based method has been proposed, which enables simultaneous X-ray phase identification and structure solution for multiphase polycrystalline samples. Test results from simulating calculations are encouraging.

## 1. Introduction   

Chapman *et al.* (2011 ▶) introduced a new approach of collecting single-crystal X-ray diffraction data using a hard X-ray free-electron laser light source (Emma *et al.*, 2010 ▶). The approach is known as serial femtosecond crystallography (SFX). There are two features in SFX: (i) diffraction before destruction and (ii) the ability to collect single-crystal X-ray diffraction data from polycrystalline (powder) samples. These features are very much beneficial to solving structures of membrane proteins as (i) they are radiation sensitive and (ii) it is difficult to grow sufficiently large and good crystals for conventional X-ray single-crystal structure analysis. These features may also lead to a revolution in X-ray polycrystalline (powder) analysis for non-biological samples in materials science. Recently Gati *et al.* (2014 ▶), Stellato *et al.* (2014 ▶) and Botha *et al.* (2014 ▶) have extended serial crystallography to using synchrotron radiation. This could help to significantly broaden the field of application. For crystals with a unit cell much smaller than those of proteins, many SFX snapshots may contain too few reflections to ensure unique indexing. Dejoie *et al.* (2013 ▶) proposed the use of a non-monochromatic broad-bandpass beam for serial snapshot crystallography. This enables SFX to be run in the pink-colour Laue mode, which significantly increases the number of reflections per snapshot and avoids the problem of partial reflection measurement. The simulating SFX experiment with powder crystals of the MFI-type zeolite ZSM-5 showed that the structure could be solved by conventional direct methods when the bandwidth values were higher than 3%. In the present paper, it will be shown by simulating calculations that, without taking advantage of the use of a non-monochromatic broad-bandpass beam, the structure of two zeolites TNU-9 (Gramm *et al.*, 2006 ▶) and NU-87 (Shannon *et al.*, 1991 ▶) with complexity higher than or similar to that of ZSM-5 (Kokotailo *et al.*, 1978 ▶) can be solved using a set of SFX snapshots from a mixed polycrystalline sample of TNU-9 and NU-87.

Many important materials are polycrystalline and cannot be grown as single crystals that are suitable for conventional X-ray single-crystal structure analysis. X-ray powder diffraction is thus widely used for characterizing and solving the structures of such materials. However, owing to the serious overlapping of reflections with similar diffraction angles, the ability of X-ray powder diffraction is very much limited. For example, while all the three zeolites mentioned above contain no more than 24 silicon atoms in the asymmetric unit, none of them was solvable by an *ab initio* X-ray powder method. Now, in SFX crystal grains in the polycrystalline sample are illuminated with X-rays one by one. The result will no longer be a one-dimensional powder diffraction pattern, but a set of three-dimensional single-crystal diffraction data, to which existing methods of single-crystal diffraction analysis may be applied. In contrast to conventional *ab initio* X-ray powder methods which are only capable of solving structures containing less than 100 atoms in the asymmetric unit, with polycrystalline samples in SFX it will not be difficult to solve structures containing more than 1000 independent atoms using *ab initio* single-crystal methods. Furthermore, in SFX, diffraction snapshots are first examined individually. Snapshots from amorphous impurities, clusters of crystals and crystals with non-merohedral twinning are not indexable and will thus be excluded. Unlike conventional X-ray powder structure analysis, SFX does not require ‘pure’ single-phase samples. Snapshots from different crystalline components in the sample can easily be identified and grouped according to the volume of their primitive unit cells determined during indexing. This opens up the possibility of simultaneous phase identification and structure solution for an unknown sample using the same set of SFX snapshots.

Zeolites are commonly used as adsorbents and catalysts in industry. They occur naturally but are also produced artificially. Structure determination of zeolites is essential for understanding their properties. Unfortunately, zeolites are polycrystalline and their structures are often too complex to solve by *ab initio* X-ray powder methods. The zeolite TNU-9 is one of the most crystallographically complex zeolites known to date. Its structure contains 24 independent Si and 52 independent O atoms. Conventional X-ray powder diffraction failed to index the diffraction pattern. A combination of diffraction phases from a few electron micrographs and X-ray powder diffraction intensities managed to locate the 24 independent Si atoms by an iterative calculation running on a Mac G5 Xserve for 16 d. The zeolite NU-87 may occur as an impurity in TNU-9 as stated by Hong *et al.* (2007 ▶). The structure of NU-87 was solved by the combination of electron diffraction and X-ray powder diffraction with the restraint of an assumed structure model. As will be seen in the following simulation, individual snapshots produced by an SFX experiment with a mixture of TNU-9 and NU-87 can be indexed and then the whole set of snapshots can be grouped into two subsets corresponding to TNU-9 and NU-87. Their crystal structure (including all Si and O atoms) can be solved automatically within half an hour by *SHELXD* (Sheldrick, 2008 ▶) running on a Mac Book Pro computer.

## 2. SFX experiment   

We simulated an SFX experiment with a non-biological polycrystalline sample. In SFX experiments, single crystals or particles in the powder sample are delivered serially as a dilute suspension by a liquid jet and flow perpendicular to the pulsed X-ray FEL beam. We assume that in most cases an X-ray pulse hits only one crystal/particle or no crystal/particle at all. We assume also that the sample consists of mainly nanocrystals of the zeolites TNU-9 and NU-87. Apart from them, the sample may contain amorphous impurities and crystalline clusters. We do not assume inclusion of twinning TNU-9 or NU-87 crystals. The reason is that no occurrence of twinning has been reported for TNU-9 and NU-87 during the solution of their structures. Concerning the dimension range of crystal grains used in the simulation, it should be small enough to exclude possibilities of doing conventional single-crystal diffraction experiments. More details are given in the next section.

## 3. Simulation of diffraction snapshots   

The simulation and processing of SFX snapshots were performed using the program suite *CrystFEL* (White *et al.*, 2012 ▶). Individual snapshots from amorphous impurities, clustering crystals or two or more randomly oriented single crystals are not indexable by *CrystFEL* and are hence rejected by the indexing process in a real experiment. Consequently, given the sample defined in the previous section, we should consider here only those diffraction snapshots from individual single crystals of TNU-9 or NU-87. 

**Figure 1:**
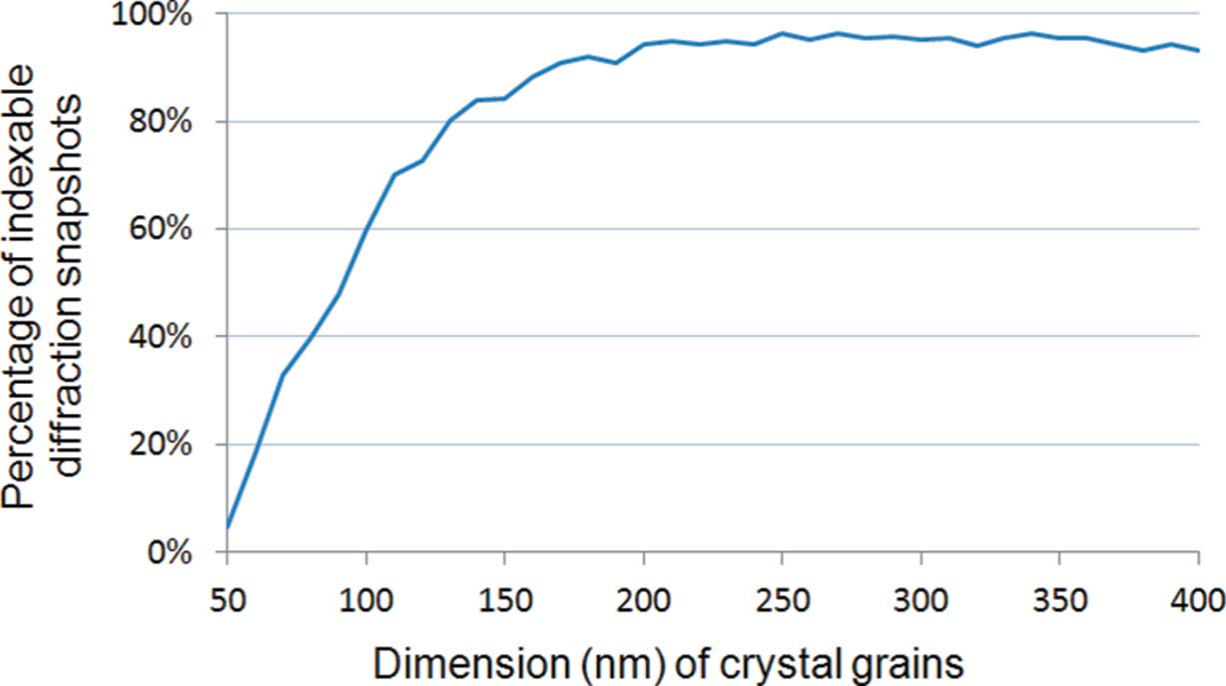
Percentage of indexable snapshots plotted against the dimension of crystal grains (counted from 1000 diffraction snapshots of the zeolite TNU-9).

Crystallographic data of TNU-9 and NU-87 are summarized in Table 1 ▶. All the parameters listed in Table 1 ▶ except the chemical compositions were assumed unknown during the test of phase identification and structure solution. Atomic parameters of the structure model of TNU-9 and NU-87 were taken from the original publications to calculate the structure factors using *gen-sfs* in *CrystFEL*. Then SFX snapshots of TNU-9 and NU-87 were simulated separately using the program *pattern_sim* in *CrystFEL*. The two sets of snapshots were mixed randomly at a ratio of TNU-9 to NU-87 equal to 4:1. The total number of snapshots used was 1 000 000 so as to allow a sufficient number of snapshots for NU-87. Conditions of the simulation are summarized in Table 2 ▶. It should be emphasized that conditions listed in Table 2 ▶, except crystal dimensions, are by no means optimized for either zeolites or non-biological small structures. They are the default in *CrystFEL* or have been used for proteins. Concerning the crystal dimensions, Schlenker & Peterson (1996 ▶) have shown that, for the zeolite ZSM-5, crystal grains of 50 unit-cell size are sufficient to produce a theoretical powder diffraction pattern, which matches very well a good quality experimental pattern. Hence, for zeolites TNU-9 and NU-87, 100 nm may be a proper value for the lower limit of the dimension range. This is also supported by Fig. 1 ▶, in which crystal dimensions are plotted against the indexing rate of simulated TNU-9 snapshots. The plot was calculated for a series of crystal dimensions and each indexing rate was counted within the corresponding 1000 TNU-9 snapshots simulated by the program *pattern_sim* in *CrystFEL* using exactly the same conditions (not including crystal dimensions) listed in Table 2 ▶. As can be seen, when the crystal dimension equals 50 nm, the indexing rate equals 4.5%. It is obviously too low to be acceptable. If the dimension equals 100 nm, then the rate increases to 60%. It is quite an acceptable value. When the dimension is equal to or greater than 200 nm, the rate is equal to or greater than 94%. We finally selected 100–300 nm as the crystal dimension range.

Summarized results on the simulation of snapshots from TNU-9 and NU 87 are listed in Table 3 ▶.

## 4. Phase identification and diffraction intensity extraction   

**Figure 2:**
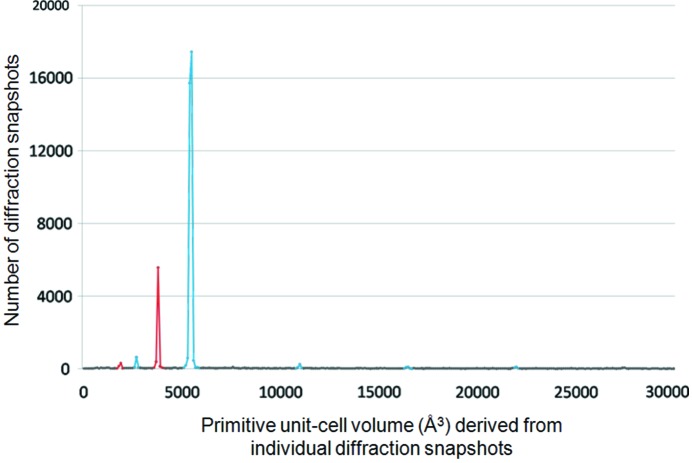
Number of diffraction snapshots plotted against the primitive unit-cell volume derived from individual diffraction snapshots.

In this section, no preliminary knowledge concerning either chemical composition or crystal structure of the sample was assumed. The critical step here is to find out how many different crystalline phases are contained in the sample. First, indexing of each diffraction snapshot was tried by the program *indexamajig* in *CrystFEL*. For those snapshots that passed through the indexing, their primitive unit-cell volume was calculated. Then numbers of snapshots were plotted against the unit-cell volumes as shown in Fig. 2 ▶. Two prominent peaks were found with the unit-cell volume located at 3800 and 5500 Å^3^. In addition to these two peaks, there are a number of much smaller peaks with their unit-cell volumes equal to *N* times or 1/*N* (*N* is a simple integer) of either 3800 or 5500 Å^3^. Peaks related to the volume of 3800 Å^3^ are coloured in red, while those related to the volume of 5500 Å^3^ are in blue. As seen, the red and blue peaks form the majority of peaks in Fig. 1 ▶. It is clear that the indexable snapshots are mainly from two different crystalline phases, the red phase and the blue phase. Averaged primitive unit-cell parameters derived by the program *indexamajig* in *CrystFEL* near the unit-cell volume at 3800 and 5500 Å^3^ are as follows:

Red phase: *a* = 13.67, *b* = 14.25, *c* = 22.37 Å, α = 89.90, β = 89.89, γ = 61.81°.

Blue phase: *a* = 17.29, *b* = 17.33, *c* = 19.49 Å, α = 88.02, β = 88.02, γ = 70.85°.

The whole set of test data was re-indexed by specifying the primitive unit-cell parameters of the red phase. 164 571 snapshots among the total 1 000 000 passed through the indexing. They were then separated to form the diffraction data set of the red phase. Similarly a set of 714 025 snapshots was separated to form the diffraction data set of the blue phase. Monte Carlo integration (Kirian *et al.*, 2010 ▶) as implemented in *CrystFEL* was applied to these two data sets separately and produced two sets of three-dimensional diffraction intensities of the red and the blue phases. Finally the program *XPREP* (http://shelx.uni-ac.gwdg.de/tutorial/english/) was applied to the two sets of three-dimensional diffraction intensities separately. The space groups were thus determined and the unit-cell parameters were converted according to crystallographic conventions. The results are as follows:

Red phase: *P*2_1_/*c*, *a* = 14.33, *b* = 22.38, *c* = 25.09 Å, β = 151.06°.

Blue phase: *C*2/*m*, *a* = 28.22, *b* = 20.08, *c* = 19.47 Å, β = 92.57°.

Comparing these results with the corresponding parameters listed in Table 1 ▶, it is clear that the red phase is NU-87, while the blue phase is TNU-9.

## 5. Structure determination   

**Figure 3:**
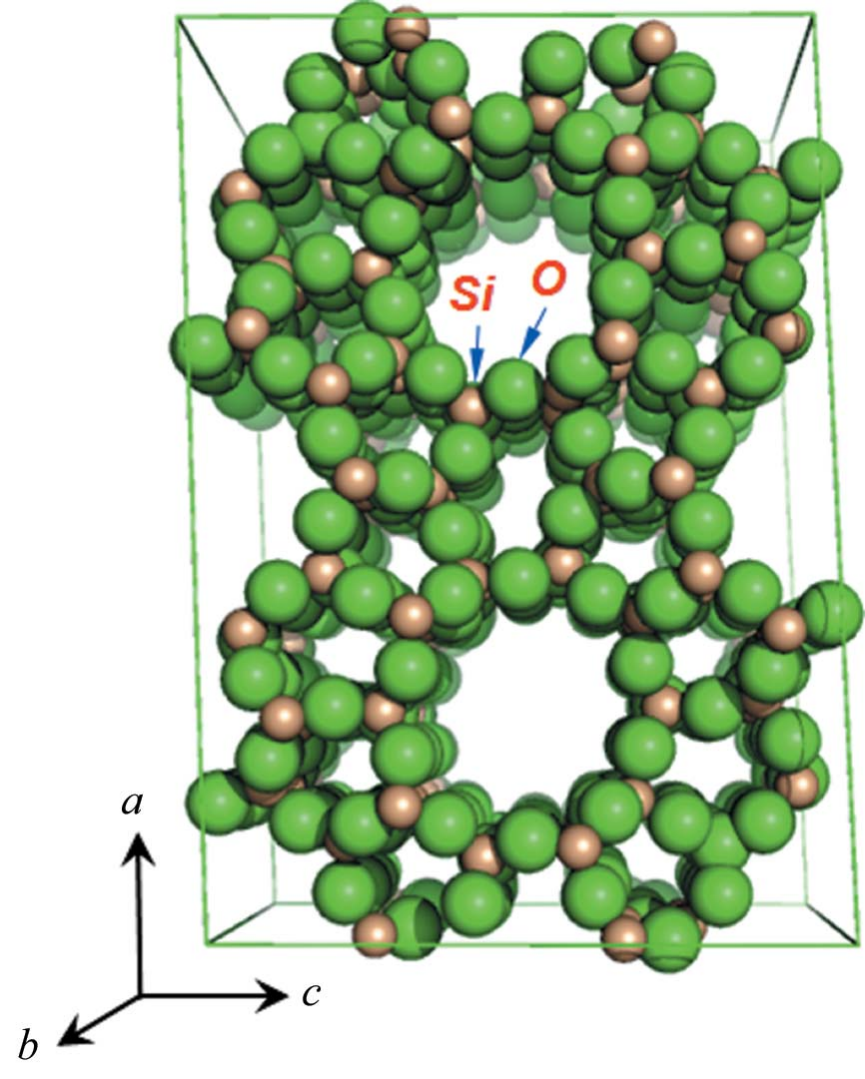
Crystal structure of TNU-9 automatically determined by *SHELXD* (plotted by *PyMOL*).

From the previous section we now have two sets of three-dimensional diffraction intensities and the corresponding space group and unit-cell parameters. Based on these, the crystal structure of the red and the blue phases can be easily solved by standard single-crystal methods. The only additional information we need is the chemical composition of the two phases (see Table 1 ▶). By using the direct methods program *SHELXD* (Sheldrick, 2008 ▶), 1000 trials under the default control reviewed the complete structure of the red phase NU-89 and the blue phase TNU-9. Summarized results are shown in Table 4 ▶. Plots of the two structures by *PyMOL* (Version 1.5.0.4, Schrödinger, LLC) are shown in Figs. 3 ▶ and 4 ▶.

## 6. Concluding remarks   

The proposed method is capable of simultaneous phase identification and structure solution for an unknown multiphase polycrystalline sample, while this is simply impossible for conventional X-ray powder diffraction. The proposed method has solved the structure of the zeolite TNU-9 easily knowing in advance only the chemical composition and diffraction intensities, which is also impossible by conventional X-ray powder diffraction. While the test in this paper was done on a simulated SFX data set, it is reasonable to expect that the serial crystallographic (SX) technique based on a third-generation synchrotron radiation source can do the same, since Stellato *et al.* (2014 ▶) and Botha *et al.* (2014 ▶) showed that it is possible to collect three-dimensional single-crystal diffraction intensities by the SX technique even for a polycrystalline protein sample.

## Figures and Tables

**Figure 4:**
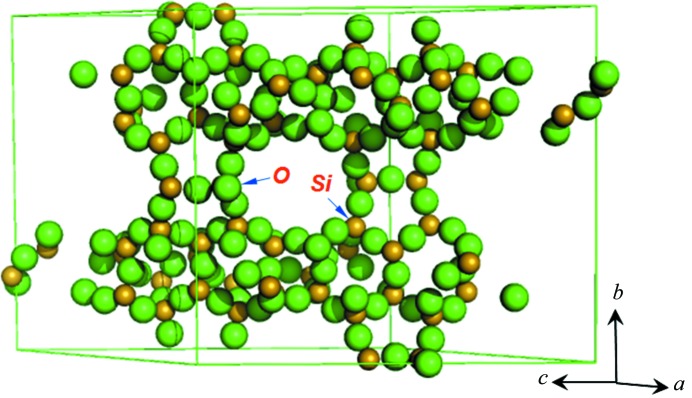
Crystal structure of NU-87 automatically determined by *SHELXD* (plotted by *PyMOL*).

**Table 1:** Summarized crystallographic data of TNU-9 and NU-87

	TNU-9	NU-87
Space group	*C*2/*m*	*P*2_1_/*c*
Unit-cell parameters		
*a* ()	28.204	14.324
*b* ()	20.043	22.376
*c* ()	19.480	25.092
()	92.30	151.51
Chemical composition of the asymmetric unit	Si_24_O_52_	Si_17_O_34_
Atomic coordinates	See the corresponding reference below:	
Reference	Gramm *et al.* (2006 ▶)	Shannon *et al.* (1991 ▶)

**Table 2:** Conditions for simulating calculations of SFX diffraction snapshots

Photon energy (eV)	2.48 10^4^
Pulse fluence (photonsm^2^)	7.073510^11^
Beam bandwidth	0.001
Beam divergence (radians)	0.001
Number of pixels on the detector	1456 1456
Pixel size (m)	110
Sample-to-detector distance (cm)	8
Dimension (nm) of crystal grains	100300
Averaged Poisson noise (applied by default of *CrystFEL*)	5.3%

**Table 3:** Summarized results of simulated snapshots of TNU-9 and NU-87

	TNU-9	NU-87
Number of patterns simulated	800000	200000
Number of patterns indexed	714025	164571
Resolution ()	19.4640.540	12.5450.547
Number of total reflections	35940	24848
Multiplicity (overall)	727	132
*R*splite (overall)	0.268	0.405

**Table 4:** Summarized results of crystal structure determination of TNU-9 and NU-87 by the direct methods program *SHELXD*

	TNU-9	NU-87
Final CC from *SHELXD* (1000 trials)	84.44	72.91
*R* factor of the *SHELXD* structure model	0.24	0.27
Error () in atomic positions		
Averaged	0.102	0.082
Maximum	0.207	0.199
